# IntelliGO: a new vector-based semantic similarity measure including annotation origin

**DOI:** 10.1186/1471-2105-11-588

**Published:** 2010-12-01

**Authors:** Sidahmed Benabderrahmane, Malika Smail-Tabbone, Olivier Poch, Amedeo Napoli, Marie-Dominique Devignes

**Affiliations:** 1 LORIA (CNRS, INRIA, Nancy-Université), Équipe Orpailleur, Bâtiment B, Campus scientifique, 54506 Vandoeuvre-lès-Nancy Cedex, France; 2 L.B.G.I., CNRS UMR7104, IGBMC, 1 rue Laurent Fries, 67404 Illkirch Strasbourg, France

## Abstract

**Background:**

The Gene Ontology (GO) is a well known controlled vocabulary describing the *biological process*, *molecular function *and *cellular component *aspects of gene annotation. It has become a widely used knowledge source in bioinformatics for annotating genes and measuring their semantic similarity. These measures generally involve the GO graph structure, the information content of GO aspects, or a combination of both. However, only a few of the semantic similarity measures described so far can handle GO annotations differently according to their origin (*i.e*. their evidence codes).

**Results:**

We present here a new semantic similarity measure called *IntelliGO *which integrates several complementary properties in a novel vector space model. The coefficients associated with each GO term that annotates a given gene or protein include its information content as well as a customized value for each type of GO evidence code. The generalized cosine similarity measure, used for calculating the dot product between two vectors, has been rigorously adapted to the context of the GO graph. The *IntelliGO *similarity measure is tested on two benchmark datasets consisting of KEGG pathways and Pfam domains grouped as clans, considering the GO *biological process *and *molecular function *terms, respectively, for a total of 683 yeast and human genes and involving more than 67,900 pair-wise comparisons. The ability of the *IntelliGO *similarity measure to express the biological cohesion of sets of genes compares favourably to four existing similarity measures. For inter-set comparison, it consistently discriminates between distinct sets of genes. Furthermore, the *IntelliGO *similarity measure allows the influence of weights assigned to evidence codes to be checked. Finally, the results obtained with a complementary reference technique give intermediate but correct correlation values with the sequence similarity, Pfam, and Enzyme classifications when compared to previously published measures.

**Conclusions:**

The *IntelliGO *similarity measure provides a customizable and comprehensive method for quantifying gene similarity based on GO annotations. It also displays a robust set-discriminating power which suggests it will be useful for functional clustering.

**Availability:**

An on-line version of the *IntelliGO *similarity measure is available at: http://bioinfo.loria.fr/Members/benabdsi/intelligo_project/

## 1 Background

### 1.1 Gene annotation

The Gene Ontology (GO) has become one of the most important and useful resources in bioinformatics [1]. This ontology of about 30,000 terms is organized as a controlled vocabulary describing the *biological process *(BP), *molecular function *(MF), and *cellular component *(CC) aspects of gene annotation, also called GO aspects [2]. The GO vocabulary is structured as a rooted Directed Acyclic Graph (rDAG) in which GO terms are the nodes connected by different hierarchical relations (mostly *is_a *and *part_of *relations). The *is-a *relation describes the fact that a given child term is a specialization of a parent term, while the *part-of *relation denotes the fact that a child term is a component of a parent term. Another GO relation *regulates *expresses the fact that one process directly affects the manifestation of another process or quality [3]. However, this relation is not considered in most studies dealing with semantic similarity measures. By definition, each rDAG has a unique root node, relationships between nodes are oriented, and there are no cycles, *i.e*. no path starts and ends at the same node.

The GO Consortium regularly updates a GO Annotation (GOA) Database [4] in which appropriate GO terms are assigned to genes or gene products from public databases. GO annotations are widely used for data mining in several bioinformatics domains, including gene functional analysis of DNA microarrays data [5], gene clustering [6-8], and semantic gene similarity [9].

It is worth noting that each GO annotation is summarized by an evidence code (EC) which traces the procedure that was used to assign a specific GO term to a given gene [10]. Out of all available ECs, only the Inferred from Electronic Annotation (IEA) code is not assigned by a curator. Manually assigned ECs fall into four general categories (see Section 2.4.3 and Table 1): author statement, experimental analysis, computational analysis, and curatorial statements. The author statement (Auth) means that the annotation either cites a published reference as the source of information (TAS for Traceable Author Statement) or it does not cite a published reference (NAS for Non traceable Author Statement). An experimental (Exp) annotation means that the annotation is based on a laboratory experiment. There are five ECs which correspond to various specific types of experimental evidence (IDA, IPI, IMP, IGI, and IEP; see Table 1 for details), plus one non specific parent code which is simply denoted as Exp. The use of an Exp EC annotation is always accompanied by the citation of a published reference. A Comp means that the annotation is based on computational analysis performed under the supervision of a human annotator. There are six types of Comp EC which correspond to various specific computational analyses (ISS, RCA, ISA, ISO, ISM and IGC; see Table 1 for details). The curatorial statement (Cur) includes the IC (Inferred by Curator) code which is used when an annotation is not supported by any direct evidence but can be reasonably inferred by a curator from other GO annotations for which evidence is available. For example, if a gene product has been annotated as a transcription factor on some experimental basis, the curator may add an IC annotation to the cellular component term *nucleus*. The ND (No biological Data available) code also belongs to the Cur category and means that a curator could not find any biological information. In practice, annotators are asked to follow a detailed decision tree in order to qualify each annotation with the proper EC [11]. Ultimately, a reference can describe multiple methods, each of which provides evidence to assign a certain GO term to a particular gene product. It is therefore common to see multiple gene annotations with identical GO identifiers but different ECs.

**Table 1:** EC weight lists assigned to the 16 GO ECs considered in this study

	Auth		Exp								Comp				Cur		Auto
**EC**	**TAS**	**NAS**	**EXP**	**IDA**	**IPI**	**IMP**	**IGI**	**IEP**	**ISS**	**RCA**	**ISA**	**ISO**	**ISM**	**IGC**	**IC**	**ND**	**IEA**

List1									1								

List2	1	0.5	0.8	0.8	0.8	0.8	0.8	0.8	0.6	0.6	0.6	0.6	0.6	0.6	0.5	0	0.4

List3	1	0.5	0.8	0.8	0.8	0.8	0.8	0.8	0.6	0.6	0.6	0.6	0.6	0.6	0.5	0	0

List4	0																1

Table 1: The various weights assigned to the ECs are listed in the following lines as EC weight lists 1 to 4. TAS: Traceable Author Statement; NAS: Non-traceable Author Statement; EXP: Inferred from Experiment; IDA: Inferred from Direct Assay; IPI: Inferred from Physical Interaction; IMP: Inferred from Mutant Phenotype; IGI: Inferred from Genetic Interaction; IEP: Inferred from Expression Pattern; ISS: Inferred from Sequence Similarity; RCA: Inferred from Reviewed Computational Analysis; ISA: Inferred from Sequence Alignment; ISO: Inferred from Sequence Orthology; ISM: Inferred from Sequence Model; IGC: Inferred from Genomic Context; IC: Inferred from Curator; IEA: Inferred from Electronic Annotation; ND: No biological Data available. The EC categories are indicated in the first line of the table. Auth: Author statement; Exp: Experimental; Comp: Computational Analysis; Cur: Curator statement; Auto: Automatically assigned.

**Figure 1:**
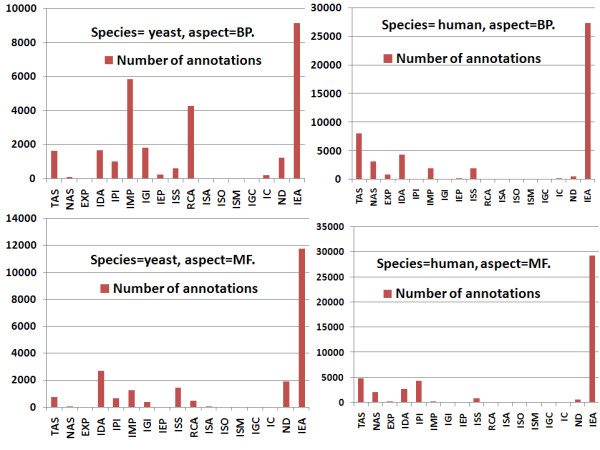
**Distribution of EC (evidence codes) in yeast and human gene annotations according to BP and MF aspects**. The number of annotations assigned to a gene with a given EC is represented for each EC. Note that some genes can be annotated twice with the same term but with a different EC. The cumulative numbers of all non-IEA annotations are 18,496 and 9,564 for the yeast BP and MF annotations, respectively, and 21,462 and 16,243 for the human BP and MF annotations, respectively. Statistics are derived from the NCBI annotation file, version June 2009.

The statistical distribution of gene annotations with respect to the various ECs is shown in Figure 1 for human and yeast BP and MF aspects. This figure shows that IEA annotations are clearly dominant in both species and for all GO aspects, but that some codes are not represented at all (e.g. ISM, IGC).

However, the ratio between non-IEA and IEA annotations is different in yeast and human. It is about 2.0 and 0.8 for the yeast BP and MF annotations compared to about 0.8 and 0.6 for the corresponding human annotations, respectively. This observation reflects a higher contribution of non-IEA annotation in yeast and is somewhat expected because of the smaller size of yeast genome and because more experiments have been carried out on yeast. In summary, GO ECs add high value to gene annotations because they trace annotation origins. However, apart for the G-SESAME and *SimGIC *measures which select GO annotations on the basis of ECs [12], only a few of the gene similarity measures described so far can handle GO annotations differently according to their ECs [9], [13]. Hence, one objective of this paper is to introduce a new semantic similarity measure which takes into account GO annotations and their associated ECs.

### 1.2 Semantic similarity measure

#### 1.2.1 The notion of semantic similarity measure

Using the general notion of similarity to identify objects which share common attributes or characteristics appears in many contexts such as word sense disambiguation, spelling correction, and information retrieval [14,15]. Similarity methods based on this notion are often called *featural approaches *because they assume that items are represented by lists of features which describe their properties. Thus, a similarity comparison involves comparing the feature lists that represent the items [16].

A similarity measure is referred to as *semantic *if it can handle the relationships that exist between the features of the items being compared. Comparing documents described by terms from a thesaurus or an ontology typically involves measuring semantic similarity [17]. Authors such as Resnik [18] or Jiang and Conrath [19] are considered as pioneers in ontology-based semantic similarity measures thanks to their long investigations in general English linguistics [20]. A general framework for comparing semantic similarity measures in a subsumption hierarchy has been proposed by Blanchard *et al*. [15]. For these authors, *tree-based similarities *fall into two large categories, namely those which only depend on the hierarchical relationships between the terms [21] and those which incorporate additional statistics such as term frequency in a corpus [22].

In the biological domain, the term *functional similarity *was introduced to describe the similarity between genes or gene products as measured by the similarity between their GO functional annotation terms. Biologists often need to establish functional similarities between genes. For example, in gene expression studies, correlations have been demonstrated between gene expression and GO semantic similarities [23,24]. Because GO terms are organized in a rDAG, the functional similarity between genes can be calculated using a semantic similarity measure. In a recent review, Pesquita *et al*. define a semantic similarity measure as a function that, given two individual ontology terms or two sets of terms annotating two biological entities, returns a numerical value reflecting the closeness in meaning between them [9]. These authors distinguish the comparison between two ontology terms from the comparison between two sets of ontology terms.

#### 1.2.2 Comparison between two terms

Concerning the comparison between individual ontology terms, the two types of approaches reviewed by Pesquita *et al*. [9] are similar to those proposed by Blanchard *et al*. [15], namely the *edge-based *measures which rely on counting edges in the graph, and *node-based *measures which exploit information contained in the considered term, its descendants and its parents.

In most *edge-based *measures, the *Shortest Path-Length *(SPL) is used as a distance measure between two terms in a graph. This indicator was used by Rada *et al*. [25] on MeSH (Medical Subject Headings) terms and by Al-Mubaid *et al*. [26] on GO terms. However, Pesquita *et al*. question whether SPL-based measures truly reflect the semantic closeness of two terms. Indeed these measures rely on two assumptions that are seldom true in biological ontologies, namely that nodes and edges are uniformly distributed, and that edges at the same level in a hierarchy correspond to the same semantic distance between terms. *Node-based *measures are probably the most cited semantic similarity measures. These mainly rely on the information content (IC) of the two terms being compared and of their closest common ancestor [18,22]. The information content of a term is based on its frequency, or probability, of occurring in a corpus. Resnik uses the negative logarithm of the probability of a term to quantify its information content, *IC*(*c_i_*) = *-Log*(*p*(*c_i_*)) [18,27]. Thus, a term with a high probability of occurring has a low IC. Conversely, very specific terms that are less frequent have a high IC. Intuitively, IC values increase as a function of depth in the hierarchy. Resnik's similarity measure between two terms consists of determining the IC of all common ancestors between two terms and selecting the maximal value, *i.e*. the IC of the most specific (i.e. lowest) common ancestor (LCA). In other words, if two terms share an ancestor with a high information content, they are considered to be semantically very similar. Since the maximum of this IC value can be greater than one, Lin introduced a normalization term into Resnik's measure yielding [22]:

(1) $$SIM_{Lin}(c_{i},c_{j})=2*\frac{IC(LCA(c_{i},c_{j}))}{IC(c_{i})+IC(c_{j})}.$$

Recently, Schlicker *et al*. improved Lin's measure by using a correction factor based on the probability of occurrence of the *LCA*. Indeed, a general ancestor should not bring too high a contribution to term comparison [28]. A limitation of *node-based *measures is that they cannot explicitly take into account the distance separating terms from their common ancestor [9]. Hybrid methods also exist which combine *edge-based *and *node-based *methods, such as those developed by Wang *et al*. [29] and Othman *et al*. [30].

#### 1.2.3 Comparison between sets of terms

Concerning the comparison between sets of terms, the approaches reviewed by Pesquita *et al*. fall into two broad categories: *pairwise *methods which simply combine the semantic similarities between all pairs of terms, and *groupwise *methods which consider a set of terms as a mathematical set, a vector, or a graph. The various *pairwise *methods differ in the strategies chosen to calculate the pairwise similarity between terms and in how pairwise similarities are combined. These methods have been thoroughly reviewed previously [9]. Hence we concentrate here on two representative examples that we chose for comparison purposes, namely the Lord measure which uses the *node-based *Resnik measure in the pairwise comparison step, and the Al-Mubaid measure which uses an *edge-based *measure. The study by Lord *et al*. in 2003 [2] provides the first description of a semantic similarity measure for GO terms. Semantic similarity between proteins is calculated as the average of all pairwise Resnik similarities between the corresponding GO annotations. In contrast, the measure defined by Al-Mubaid *et al*. [26], [31] considers the shortest path length (SPL) matrix between all pairs of GO terms that annotate two genes or gene products. It then calculates the average of all SPL values in the matrix, which represents the path length between two gene products. Finally, a transfer function is applied to the average SPL to convert it into a similarity value (see Methods). In *group-wise *methods, non semantic similarity measures co-exist with semantic ones. For example, the early Jaccard and Dice methods of counting the percentage of common terms between two sets are clearly non semantic [15]. However, in subsequent studies, various authors used sets of GO terms that have been extended with all term ancestors [32], [33].

Graph-based similarity measures are currently implemented in the Bioconductor GOstats package [34]. Each protein or gene can be associated with a graph which is induced by taking the most specific GO terms annotating the protein, and by finding all parents of those terms up to the root node. The union-intersection and longest shared path (*SimUI *) method can be used to calculate the between-graph similarity, for example. This method was tested by Guo *et al*. on human regulatory pathways [35]. Recently, the *SimGIC *method was introduced to improve the *SimUI *method by weighting terms with their information content [36].

Finally, vector-based similarity measures need to define an *annotation Vector-Space Model *(VSM) by analogy to the classical VSM described for document retrieval [37], [38], [39]. In the *annotation *VSM, each gene is represented by a vector $\vec{g}$ in a *k*-dimensional space constructed from basis vectors ${\vec{e}}_{i}$ which correspond to the *k *annotation terms [40,41]. Thus, text documents and terms are replaced by gene and annotation terms, respectively, according to

(2) $$\vec{g}=\sum_{i}\alpha_{i}*{\vec{e}}_{i},$$

where ${\vec{e}}_{i}$ is the *i*-th basis vector in the VSM *annotation *corresponding to the annotation term *t_i_*, and where *α_i _*is the coefficient of that term.

The DAVID tool, which was developed for functional characterization of gene clusters [6], uses this representation with binary coefficients which are set to 1 if a gene is annotated by a term and zero otherwise. Similarity is then calculated using "Kappa statistics" [42] which consider the significance of observed co-occurrences with respect to chance. However, this approach does not take into account the semantic similarity between functional annotation terms. In another study by Chabalier *et al*., the coefficients are defined as weights corresponding to the information content of each annotation term. The similarity between two genes is then computed using a cosine similarity measure. The semantic feature in Chabalier's method consists of a pre-filtering step which retains only those GO annotations at a certain level in the GO graph.

Ganesan *et al*. introduced a new vector-based semantic similarity measure in the domain of information retrieval [14]. When two annotation terms are different, this extended cosine measure allows the dot product between their corresponding vectors to be non-zero, thus expressing the semantic similarity that may exist between them. In other words, the components of the vector space are not mutually orthogonal. We decided to use this approach in the context of GO annotations. Hence the *IntelliGO *similarity measure defines a new vector-based representation of gene annotations with meaningful coefficients based on both information content and annotation origin. Vector comparison is based on the extended cosine measure and involves an *edge-based *similarity measure between each vector component.

## 2 Results

### 2.1 The IntelliGO Vector Space Model to represent gene annotations

#### 2.1.1 The IntelliGO weighting scheme

The first originality of the *IntelliGO *VSM lies in its weighting scheme. The coefficients assigned to each vector component (GO term) are composed of two measures analogous to the *tf-idf *measures used for document retrieval [43]. On one hand, a weight *w*(*g*, *t_i_*) is assigned to the EC that traces the annotation origin and qualifies the importance of the association between a specific GO term *t_i _*and a given gene *g*. On the other hand, the *Inverse Annotation Frequency *(*IAF *) measure is defined for a given corpus of annotated genes as the ratio between the total number of genes *G_Tot _*and the number of genes $G_{t_{i}}$ annotated by the term *t_i_*. The *IAF *value of term *t_i _*is calculated as

(3) $$IAF(t_{i})=log\frac{G_{Tot}}{G_{t_{i}}}.$$

This definition is clearly related to what was defined above as the information content of a GO term in an annotation corpus. It can be verified that GO terms which are frequently used to annotate genes in a corpus will display a low *IAF *value, whereas GO terms that are rarely used will display a high *IAF *which reflects their specificity and their potentially high contribution to vector comparison. In summary, the coefficient *α_i _*is defined as

(4) $$\alpha_{i}=w(g,\;t_{i})*IAF(t_{i}).$$

#### 2.1.2 The IntelliGO generalized cosine similarity measure

The second innovative feature of the *IntelliGO *VSM concerns the basis vectors themselves. In classical VSMs, the basis is orthonormal, i.e. the base vectors are normed and mutually orthogonal. This corresponds to the assumption that each dimension of the vector space (here each annotation term) is independent from the others. In the case of gene annotation, this assumption obviously conflicts with the fact that GO terms are interrelated in the GO rDAG structure. Therefore, in the *IntelliGO *VSM, basis vectors are not considered as orthogonal to each other within a given GO aspect (BP, MF, or CC).

A similar situation has been handled by Ganesan *et al*. [14] in the context of document retrieval using a tree-hierarchy of indexating terms. Given two annotation terms, *t_i _*and *t_j_*, represented by their vectors, ${\vec{e}}_{i}$ and ${\vec{e}}_{j}$, respectively, the *Generalized Cosine-Similarity Measure *(GCSM) defines the dot product between these two base vectors as

(5) $${\vec{e}}_{i}*{\vec{e}}_{j}=2*\frac{Depth(LCA(t_{i},t_{j}))}{Depth(t_{i})+Depth(t_{j})}.$$

The GCSM measure has been applied successfully by Blott *et al*. to a corpus of publications indexed using MeSH terms [43]. However applying the GCSM to the GO rDAG is not trivial. As mentioned above, in an rDAG there exist more than one path from one term to the *Root*. This has two consequences for the GCSM formula (5). Firstly, there may exist more than one LCA for two terms. Secondly, the depth value of a term is not unique but depends on the path which is followed up to the rDAG root. We therefore adapted the GCSM formula to rDAGs in a formal approach inspired by Couto *et al*. [44].

The GO controlled vocabulary can be defined as a triplet *γ *= (*T*, Ξ, *R*), where *T *is the set of annotation terms, Ξ is the set of the two main hierarchical relations that may hold between terms, i.e. Ξ = {*is-a, part-of *}. The third element *R *contains a set of triples τ = (*t*, *t*', ξ), where *t*, *t*' ∈ *T *, ξ ∈ Ξ and *t*ξ*t*'. Note that ξ is an oriented child-parent relation and that ∀ τ ∈ *R*, the relation ξ between *t *and *t*' is either *is-a *or *part-of*. In the *γ *vocabulary, the *Root *term represents the top-level node of the GO rDAG. Indeed, *Root *is the direct parent of three nodes, *BiologicalProcess*, *CellularComponent*, and *MolecularFunction*. These are also called aspect-specific roots. The *Root *node does not have any parents, and hence the collection *R *does not contain any triple in which *t *= *Root*. All GO terms in *T *are related to the root node through their aspect-specific root. Let *Parents *be a function that returns the set of direct parents of a given term *t*:

(6) $$\begin{array}{c} Parents:T\text{​}\to P(T),\\ \begin{array}{ccc} Parents(t)=\{t^{\prime }\in T & |\exists \xi \in \Xi ,\exists \tau \in R, & \tau =(t,t^{\prime },\xi )\}, \end{array} \end{array}$$

where P(*T *) refers to the set of all possible subsets of *T*. Note that *Parents*(*Root*) = ∅. The function *Parents *is used to define the *RootPath *function as the set of directed paths descending from the *Root *term to a given term *t*:

(7) $$\begin{array}{l} RootPath:\;T\to P(P(T)),\\ RootPath(t)=\{\begin{array}{cc} \begin{array}{l} \{\{Root\}\}ift=Root\\ \left\{\{t_{1},\;\ldots ,\;t_{n}\}\right|\\ (t_{1}=Root)\wedge \\ (t_{n}=t)\wedge \\ \left(t_{n-1}\in Parents(t_{n})\right)\\ \wedge (\{t_{1},\;\ldots ,\;t_{n-1}\}\in \\ RootPath\;(t_{n-1}))\}\text{otherwise} \end{array} & \cdot \end{array} \end{array}$$

Thus, each path between the *Root *term and a term *t *is a set of terms Φ ∈ *RootPath*(*t*).

The length of a path separating a term *t *from the *Root *term is defined as the number of edges connecting the nodes in the path, and is also called the *Depth *of term *t*. However, due to the multiplicity of paths in rDAG, there can be more than one depth value associated with a term. In the following, and by way of demonstration, we define *Depth*(*t*) as the function associating a term *t *with its maximal depth:

(8) $$Depth(t)=Max_{i}(|\Phi_{i}|)-1|\Phi_{i}\in RootPath(t).$$

Note that since *RootPath*(*Root*) = {{Root}}, we have *Depth*(*Root*) = |{Root}| -1 = 0.

We then define the *Ancestors *function to identify an ancestor term of a given term *t *as any element *α *of a path Φ ∈ *RootPath*(*t*).

Ancestors: T→P(T)

and

(9) Ancestors(t)={α∈T|∃Φ, (Φ∈RootPath(t))∧(α∈Φ)}.

Thus, the common ancestors of two terms *t_a _*and *t_b _*can be defined as:

(10) $$CommonAnc(t_{a},\;t_{b})=Ancestors(t_{a})\cap Ancestors(t_{b}).$$

Let *LCAset*(*t_a_*, *t_b_*) be the set of lowest common ancestors of terms *t_a_*, *t_b_*. The lowest common ancestors are at the maximal distance from the root node. In other words their depth is the maximum depth of all terms *α *∈ *CommonAnc*(*t_a_*, *t_b_*). Note that this value is unique but it may correspond to more than one *LCA *term:

LCAset: TxT→P(T),

(11) $$\begin{array}{l} LCAset\;(t_{a},\;t_{b})=\{\alpha \in CommonAnc(t_{a},\;t_{b})\;|\\ Depth(\alpha )=Max_{i}(Depth(a_{i})),\\ a_{i}\in CommonAnc(t_{a},\;t_{b})\}. \end{array}$$

Having defined the *LCAset*, it is possible to define a subset of paths from the *Root *term to a given term *t *that pass through one of the *LCA *terms and subsequently ascend to the root node using the longest path between the *LCA *and the *Root *term. This notion is called *ConstrainedRootPath*, and can be calculated for any pair (t, s) with *s *∈ *Ancestors*(*t*):

(12) $$\begin{array}{l} ConstrainedRootPath\;(t,\;s)=\{\Phi_{i}\in RootPath(t)|\\ (s\in \Phi_{i})\wedge \\ (\forall \Phi_{j},\;((\Phi_{j}\in RootPath(s))\wedge \\ (\Phi_{j}\subset \Phi_{i}))⇌(|\Phi_{j}|=Depth(s)+1))\}. \end{array}$$

This leads to a precise definition of the path length *PL_k_*(*t*, *s*), for *s *∈ *Ancestors*(*t*) and for a given path Φ*_k _*∈ *ConstrainedRootPath*(*t*, *s*) as:

(13) $$PL_{k}(t_{i},\;s)=|\Phi_{k}|-1-Depth(s).$$

For a given *LCA *∈ *LCAset*(*t_i_*, *t_j_*), we can now define the shortest path length (SPL) between two terms *t_i _*and *t_j _*passing through this lowest common ancestor as

(14) $$\begin{array}{l} SPL(t_{i},\;t_{j},\;LCA)=Min_{k}(PL_{k}(t_{i},\;LCA))+\\ Min_{h}(PL_{h}(t_{j},\;LCA)). \end{array}$$

The minimal SPL between terms *t_i _*and *t_j _*considering all their possible *LCA*s is thus given by

(15) $$\begin{array}{l} MinSPL(t_{i},\;t_{j})=Min_{l}(SPL(t_{i},\;t_{j},\;LCA_{l}))\;|\\ LCA_{l}\;\in LCAset(t_{i},\;t_{j}). \end{array}$$

Returning to the GCSM formula (5), we now relate *Depth*(*t_i_*)+*Depth*(*t_j_*) in the denominator of the expression with *MinSPL*(*t_i_*, *t_j_*) and *Depth*(*LCA*). Note that from (8) we have

*Depth*(*t_i_*) = *Max_k_*(|Φ*_k_*| - 1), with Φ*_k _*∈ *RootPath*(*t_i_*). From (13) we have

*PL_k_*(*t_i_*, *LCA*) = |Φ*_k_*| - 1 - *Depth*(*LCA*) with Φ*_k _*∈ *ConstrainedRootPath*(*t_i_*, *LCA*) and *ConstrainedRootPath*(*t_i_*, *LCA*) ⊂ *RootPath*(*t*). Given any *LCA *∈ *LCAset*(*t_i_*, *t_j_*), it is then easy to demonstrate that

(16) $$Depth(t_{i})\geq Min_{k}(PL_{k}(t_{i},\;LCA))+Depth(LCA).$$

Similarly,

(17) $$Depth(t_{j})\geq Min_{h}(PL_{h}(t_{j},\;LCA))+Depth(LCA).$$

Thus,

(18) $$\begin{array}{l} Depth\;(t_{i})+Depth(t_{j})\geq \\ SPL(t_{i},\;t_{j},\;LCA)+2*Depth(LCA)\geq \\ MinSPL(t_{i},\;t_{j})+2*Depth(LCA). \end{array}$$

In the case of a tree, this inequality becomes an equality.

The semantic similarity between two terms is assumed to be inversely proportional to the length of the path separating the two terms across their *LCA*. When we adapt the GCSM measure in (5) by replacing in the denominator the sum *Depth*(*t_i_*) + *Depth*(*t_j_*) by the smaller sum *MinSPL*(*t_i_*, *t_j_*) + 2 _* _*Depth*(*LCA*), we ensure that the dot product between two base vectors will be maximized. With this adaptation, the *IntelliGO *dot product between two base vectors corresponding to two GO terms *t_i _*and *t_j _*is defined as

(19) $${\vec{e}}_{i}*{\vec{e}}_{j}=\frac{2*Depth(LCA)}{MinSPL(t_{i},t_{j})+2*Depth(LCA)}.$$

One can verify that with this definition, the dot product takes values in the interval 0[1]. We observe that for $i=j,{\vec{e}}_{i}*{\vec{e}}_{i}=1$, since *MinSPL*(*t_i_*, *t_j_*) = 0. Moreover, when two terms are only related through the root of the rDAG, we have ${\vec{e}}_{i}*{\vec{e}}_{i}=0$ because *Depth*(*Root*) = 0. In any other case, the value of the dot product represents a non zero *edge-based *similarity between terms. Note that this value clearly depends on the rDAG structure of the GO graph.

### 2.2 The IntelliGO semantic similarity measure

In summary, the *IntelliGO *semantic similarity measure between two genes *g *and *h *represented by their vectors $\vec{g}$ and $\vec{h}$, respectively, is given by the following cosine formula:

(20) $$SIM_{IntelliGO}(g,h)=\frac{\vec{g}*\vec{h}}{\sqrt{\vec{g}*\vec{g}}\sqrt{\vec{h}*\vec{h}}},$$

where:

• $\vec{g}=\sum_{i}\alpha_{i}*{\vec{e}}_{i}$: the vectorial representation of the gene *g *in the *IntelliGO *VSM.

• $\vec{h}=\sum_{j}\beta_{j}*{\vec{e}}_{j}$: the vectorial representation of the gene *h *in the *IntelliGO *VSM.

• *α_i _*= *w*(*g, t_i_*)* *IAF*(*t_i_*) the coefficient of term *t_i _*for gene *g*, where w(*g, t_i_*) represents the weight assigned to the evidence code between *t_i _*and *g*, and *IAF*(*t_i_*) is the inverse annotation frequency of the term *t_i_*.

• *β_j _*= *w*(*h, t_j_*)* *IAF*(*t_j_*) the coefficient of term *t_j _*for gene *h*.

• ${\vec{g}}^{*}\vec{h}=\sum_{i,j}\alpha_{i}*\beta_{j}*{\vec{e}}_{i}*{\vec{e}}_{j}$: the dot product between the two gene vectors.

• ${\vec{e}}_{i}*{\vec{e}}_{j}=\frac{2*Depth(LCA)}{MinSPL(t_{i},t_{j})+2*Depth(LCA)}$: the dot product between ${\vec{e}}_{i}$ and ${\vec{e}}_{j}({\vec{e}}_{i}*{\vec{e}}_{j}\neq 0$ if the corresponding terms *t_i _*and *t_j _*share common ancestors other than the rDAG root).

### 2.3 The IntelliGO Algorithm

The *IntelliGO *algorithm was designed to calculate the similarity measure between two genes, taking as input their identifiers in the NCBI GENE database, and as parameters a GO aspect (BP, MF, CC), a particular species, and a list of weights associated with GO ECs. The output is the *IntelliGO *similarity value between the two genes. In order to calculate this efficiently, we first extract from the NCBI annotation file [45] the list of all non redundant GO terms and the list of associated genes they annotate, whatever their evidence codes. The *IAF *values are then calculated and stored in the *SpeciesIAF *file. We then construct all possible pairs of GO terms and query the AMIGO database [46] to recover their *LCA*, *Depth*(*LCA*) and *SPL *values. Each dot product between two vectors representing two GO terms can thus be pre-calculated and stored in the *DotProduct *file.

The first step of the *IntelliGO *algorithm consists of filtering the NCBI file with the user's parameters (GO aspect, species and list of weights assigned to ECs) to produce a *CuratedAnnotation *file from which all genes of species and GO aspects other than those selected are removed. If a gene is annotated several times by the same GO term with different ECs, the program retains the EC having the greatest weight in the list of EC weights given as parameter. Then, for two input NCBI gene identifiers, the *IntelliGO *function (i) retrieves from the *CuratedAnnotation *file the list of GO terms annotating the two genes and their associated ECs, (ii) calculates from the *SpeciesIAF *file and the list of EC weights, all the coefficients of the two gene representations in the *IntelliGO *VSM, (iii) constructs the pairs of terms required to calculate the similarity value between the two vectors, (iv) assigns from the *DotProduct *file the corresponding value to each dot product, and (v) finally calculates the *IntelliGO *similarity value according to (20).

### 2.4 Testing the IntelliGO semantic similarity measure

#### 2.4.1 Benchmarking datasets and testing protocol

We evaluated our method using two different benchmarks depending on the GO aspect. For the KEGG benchmark, we selected a representative set of 13 yeast and 13 human diverse KEGG pathways [47] which contain a reasonable number of genes (between 10 and 30). The selected pathways are listed in Table 2. The genes in these pathways were retrieved from KEGG using the *DBGET *database retrieval system [48]. Assuming that genes which belong to the same pathway are often related to a similar biological process, the similarity values calculated for this dataset should be related to the BP GO aspect.

**Table 2:** List of yeast and human pathways used in this study.

KEGG	KEGG	Yeast	Name	Nb genes	Human	Name	Nb genes
**Category**	**Subcategory**	**Pathway**			**Pathway**		

01100 Metabolism	01101 Carbohydrate Metabolism	sce00562	Inositol phosphate metabolism	15	hsa00040	Pentose and glucuronate interconversions	26
	
	01102 Energy Metabolism	sce00920	Sulfur metabolism	13	hsa00920	Sulfur metabolism	13
	
	01103 Lipid Metabolism	sce00600	Sphingolipid metabolism	13	hsa00140	C21-Steroid homone metabolism	17
	
	01105 Amino Acid	sce00300	Lysine biosynthesis	13	hsa00290	Valine, leucine and isoleucine biosynthesis	11
	
	Metabolism	sce00410	Alanine biosynthesis	8			
	
	01107 Glycan Biosynthesis and Metabolism	sce00514	O-Mannosyl glycan biosynthesis	13	hsa00563	Glycosylphosphatidylinositol (GPI)-anchor biosynthesis	23
	
	01109 Metabolism of Cofactors and Vitamins	sce00670	One carbon pool by folate	14	hsa00670	One carbon pool by folate	16
	
	01110 Biosynthesis of Secondary Metabolites	sce00903	Limonene and pinene degradation	7	hsa00232	Caffeine metabolism	7

01120 Genetic Information Processing	01121 Transcription	sce03022	Basal transcription factors	24	hsa03022	Basal transcription factors	38
	
					hsa03020	RNA polymerase	29
	
	01123 Folding, Sorting and Degradation	sce04130	SNARE interactionst in vesicular Transport	23	hsa04130	SNARE interactions in vesicular transport	38
	
	01124 Replication and Repair	sce03450	Non-homologous end-joining	10	hsa03450	Non-homologous end-joining	14
	
					hsa03430	Mismatch repair	23

01130 Environmental Information Processing	01132 Signal Transduction	sce04070	Phosphatidylinositol signaling system	15			

01140 Cellular Processes	01151 Transport and Catabolism	sce04140	Regulation of autophagy	17			

01160 Human Diseases	01164 Metabolic Disorders				hsa04950	Maturity onset diabetes of the young	25

Total genes number				185			280

Non-IEA:IEA ratio				572:435 (1.3)			560:620 (0.9)

Table 2: The KEGG categories and subcategories are indicated for each pathway as well as its name and the number of genes it contains (KEGG version Dec 2009). The non-IEA:IEA ratio refers to *Biological Process *GO annotation of the complete set of genes for each species.

For the Pfam benchmark, we selected a set of clans (groups of highly related Pfam entries) from the Sanger Pfam database [49]. In order to maximize diversity in the benchmarking dataset, yeast and human sequences were retrieved from the 10 different Pfam clans listed in Table 3. For each selected Pfam clan, we used all the associated Pfam entry identifiers to query the Uniprot database [50] and retrieve the corresponding human and yeast gene identifiers. Assuming that genes which share common domains in a Pfam clan often have a similar molecular function, the similarity values calculated for this second dataset should be related to the MF GO aspect.

**Table 3:** List of yeast and human genes and Pfam clans used this study.

Pfams clan accession (yeast)	Nb genes	Pfams clan name	Pfams clan accession (human)	Nb genes	Pfams clan name
CL0328.1	15	2heme_cytochrom	CL0099.10	18	ALDH-like
CL0059.12	13	6_Hairpin	CL0106.10	8	6PGD_C
CL0092.9	8	ADF	CL0417.1	9	BIR-like
CL0099.10	11	ALDH-like	CL0165.8	5	Cache
CL0179.11	11	ATP-grasp	CL0149.9	7	CoA-acyltrans
CL0255.6	7	ATP_synthase	CL0085.11	12	FAD_DHS
CL0378.1	10	Ac-CoA-synth	CL0076.9	18	FAD_Lum_binding
CL0257.6	18	Acetyltrans-like	CL0289.3	6	FBD
CL0034.12	11	Amidohydrolase	CL0119.10	7	Flavokinase
CL0135.8	14	Arrestin_N-like	CL0042.9	10	Flavoprotein

Total genes number	118			100	

Non-IEA:IEA ratio	121:366 (0.3)			144:309 (0.46)	

Table 3: Clans are indicated by their accession identifier in the Sanger Pfam database (October 2009 release) and by the number of genes retrieved either in yeast (left part) or in human (right part). Each clan contains several Pfam entries listed in the Pfam_C file at [57]. The non-IEA:IEA ratio refers to the *Molecular Function *GO annotation of the complete set of genes for each species.

For each set of genes, an *intra-set average gene similarity *was calculated as the average of all pairwise similarity values within a set of genes. In contrast, an *inter-set average gene similarity *was also calculated between two sets *S_a _*and *S_b _*as the average of all similarity values calculated for pairs of genes from each of the two sets *S_a _*and *S_b_*. A discriminating power can then be defined according to the ratio of the intra-set and inter-set average gene similarities (see Methods). We compared the values obtained with the *IntelliGO *similarity measure with the four other representative similarity measures described above, namely the Lord measure which is based on the Resnick term-term similarity, the Al-Mubaid measure which considers only the path length between GO terms [31], a standard vector-based cosine similarity measure, and the *SimGIC *measure which is one of the *graph-based *methods described above (see Section 1.2.3). For each dataset, we evaluated our measure firstly by comparing the intra-set similarity values with those obtained with other measures, and then by studying the effect of varying the list of weights assigned to the ECs. We then compared the discriminating power of the *IntelliGO *similarity measure with three other measures. We also tested our measure on a reference dataset using a recently available on-line evaluation tool.

#### 2.4.2 Intra-set similarity

**Figure 2:**
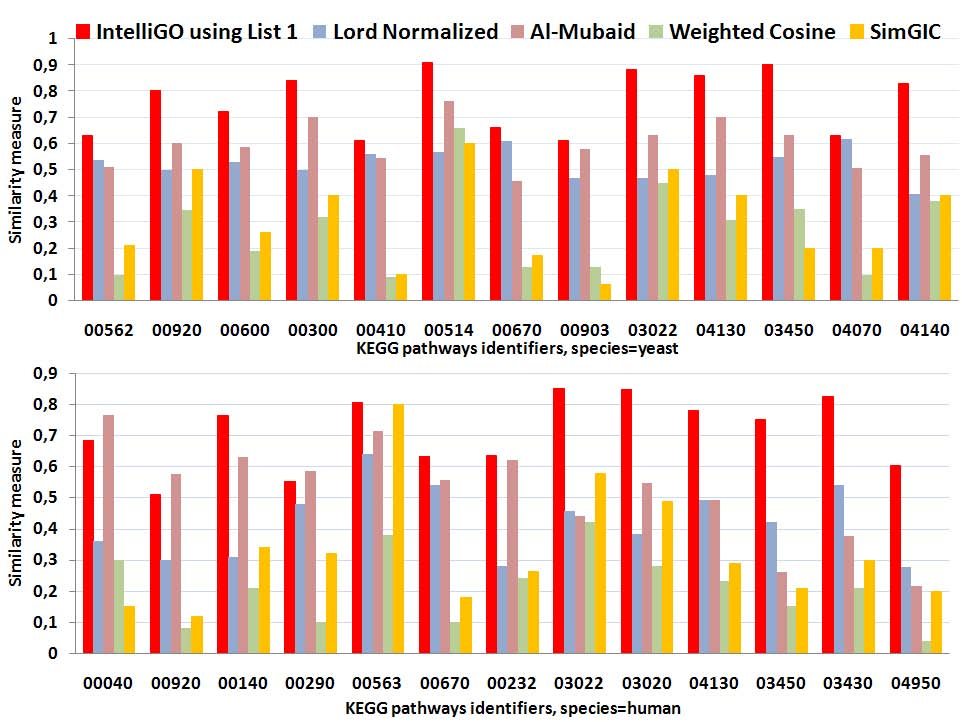
**Intra-set similarities with the KEGG pathway dataset using BP annotations**. The intra-set similarity is calculated as the mean of all pairwise gene similarities within a KEGG pathway, with the four measures compared in this study, namely, *IntelliGO *(using EC weight *List1 *), Lord-normalized, Al-Mubaid, and Weighted-cosine. A set of thirteen pathways were selected from the KEGG Pathway database for yeast (top panel) and human (bottom panel) pathways. Only BP annotations are used here (see also Table 2).

We produced all intra-set similarities with the *IntelliGO *similarity measure using EC *List1 *(all weights set to 1.0, see Table 1). We also implemented and tested four other measures, namely Lord-normalized, Al-Mubaid, the classical *weighted-cosine *measure, and the SimGIC measure (see Methods) on the same sets of genes. The results obtained with the KEGG pathways using BP annotations are shown in Figure 2. For each KEGG pathway (x-axis), the intra-set similarity values are represented as histograms (y-axis). Similarity values vary from one pathway to another, reflecting variation in the coherence of gene annotations within pathways. Variations from one pathway to another are relatively uniform for all measures except the Lord measure. For example, intra-set similarity values of the sce00410 pathway are smaller than those of sce00300 for all measures except for the Lord measure. The same is observed between pathways hsa00920 and hsa00140.

A positive feature of the *IntelliGO *similarity measure is that unlike other measures, all intra-set values are greater than or equal to 0.5. The relatively low values obtained with the *weighted-cosine *measure can be explained by the numerous null pairwise values generated by this method. This is because this measure assumes that the dimensions of the space vector are orthogonal to each other. Hence, whenever two genes lack a common annotation term their dot product is null, and so also is their similarity value. Indeed, null pairwise similarity values are observed in all pathways except for one in human and three in yeast (details not shown).

**Figure 3:**
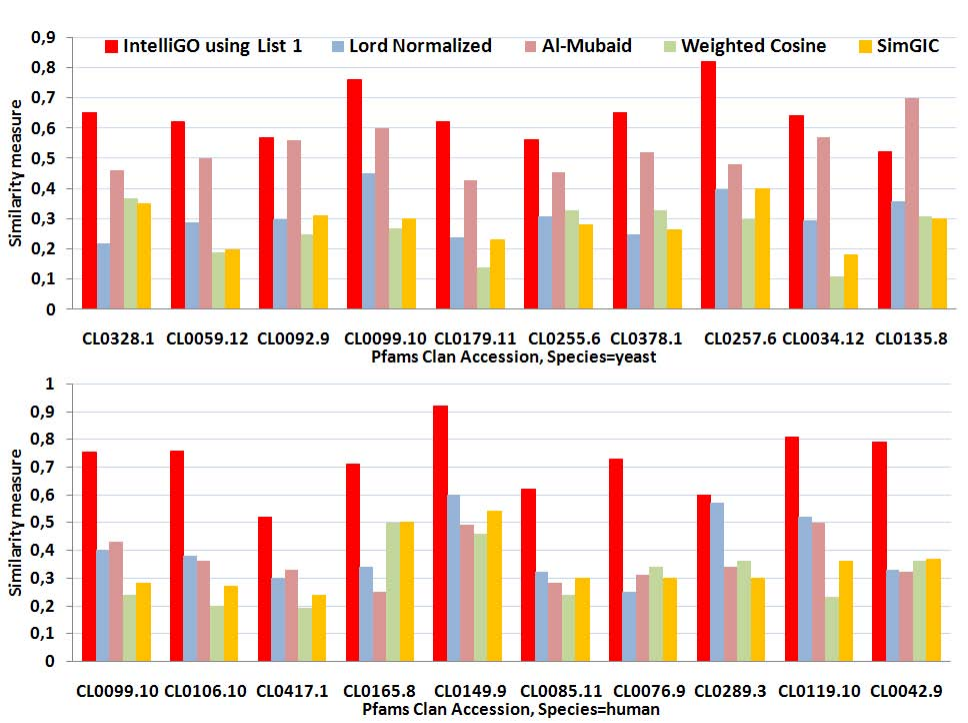
**Intra-set similarities with the Pfam clan dataset using MF annotations**. The intra-set similarity is calculated for all genes of a given species within a Pfam clan using MF annotations. Two collections of ten Pfam clans were selected from the Sanger Pfam database to retrieve yeast (top panel) and human (bottom panel) genes belonging to these clans (see also Table 3).

Very similar results were obtained with the Pfam benchmarking dataset which was analyzed on the basis of MF annotations (Figure 3). Here again, the *IntelliGO *similarity measure always provides intra-set similarity values greater than or equal to 0.5, which is not the case for the other measures. As before, the *weighted-cosine *yields the lowest intra-set similarity values for the reason explained above. This inconvenience led us to skip this measure in later stages of the work. In summary, our comparison of intra-set similarity values for two benchmarking datasets demonstrates the robustness of the *IntelliGO *similarity measure and its ability to capture the internal coherence of gene annotation within predefined sets of genes.

#### 2.4.3 Influence of EC weight lists

The second part of our evaluation is the study of the effect of varying the weights assigned to ECs in the *IntelliGO *similarity measure. As a first experiment, we used four lists of EC weights (see Table 1). In *List1*, all EC weights are equal to 1.0, which makes all ECs equivalent in their contribution to the similarity value. *List1 *was used above to compare the *IntelliGO *measure with the four other similarity measures (Figure 2 and 3) because these measures do not consider varying ECs weights in the calculation. In *List2*, the EC weights have been arbitrarily defined to represent the assumption that the *Exp *category of ECs is more reliable than the *Comp *category, and that the non-supervised *IEA *code is less reliable than *Comp *codes. *List3 *excludes *IEA *code in order to test the similarity measure when using only supervised annotations. Finally, *List4 *represents the opposite situation by retaining only the *IEA *code to test the contribution of IEA annotations.

**Figure 4:**
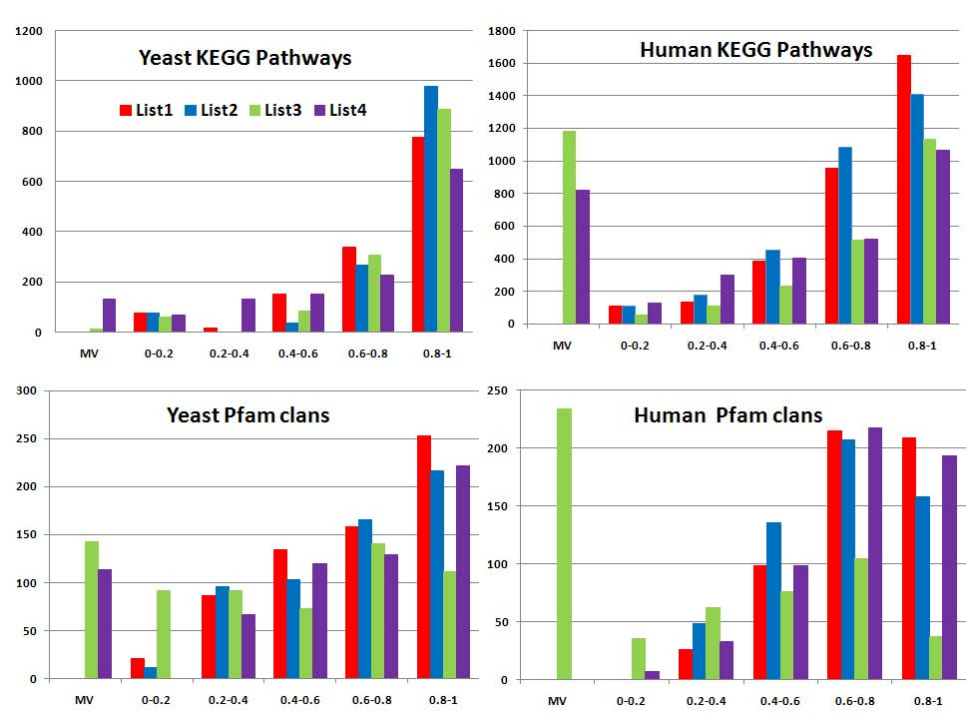
**Influence of various EC weight lists on the distribution of pairwise similarity values obtained for intra-set similarity calculation**. KEGG pathway datasets are handled with BP annotations, and Pfam clans with MF annotations. The MV bar is for *Missing Values *and represents the number of pairwise similarity values that cannot be calculated using *List3 *or *List4 *due to the missing annotations for certain genes. Pairwise similarity intervals are displayed on the × axis of the histograms, while values on the y axis represent the number of pairwise similarity values present in each interval.

These four lists were used to calculate IntelliGO intra-set similarity values on the same datasets as before. For each dataset, the distribution of all pairwise similarity values used to calculate the intra-set averages is shown in Figure 4 with each weight list being shown as a histogram with a class interval of 0.2. On the left of each histogram a *Missing Values *bar (MV) shows the number of pairwise similarity values that cannot be calculated with *List3 *or *List4 *due to the complete absence of annotations for certain genes. As expected, since intra-set similarity values with the IntelliGO measure are greater than 0.5, the highest numbers of pairwise values are found in the intervals 0.6-0.8 and 0.8-1.0 for all weight lists considered here. For *List1 *and *List2*, the distribution of values looks similar for all datasets. The effect of excluding the IEA code (*List3 *) or considering it alone (*List4 *) differs between the KEGG pathways and Pfam clans, i.e. between the BP and MF annotations. It also varies between the yeast and human datasets, reflecting the different ratios of IEA versus non-IEA annotations in these two species (see Figure 1 and Table 2 and 3). For the yeast KEGG pathways, the most striking variation is observed with *List4 *which gives a marked decrease in the number of values in the 0.8-1.0 class interval, and a significant number of missing values.

This means that for this dataset, using only IEA BP annotations yields generally lower similarity values and excludes from the calculation those genes without any IEA annotation (11 genes). This reflects the relatively high ratio (1.3) of non-IEA to IEA BP annotations in this dataset, and in yeast in general (2.0). A similar behavior is observed with the human KEGG pathways, not only with *List4 *but also with *List3*. The higher *Missing Values *bars in this dataset results from the high number of genes having either no IEA BP annotations (49 genes) or only IEA BP annotations (68 genes). This type of analysis shows that for such a dataset, IEA annotations are useful to capture intra-set pairwise similarity but they are not sufficient *per se*.

For the yeast and human Pfam clans, the distribution of values obtained with *List3 *is clearly shifted towards lower class intervals and *Missing Values *bars. This reflects the extent of the IEA MF annotations, and their important role in capturing intra-set similarity in these datasets (the ratios between non-IEA and IEA MF annotations are 0.3 and 0.46 for the yeast and human Pfam clan datasets, respectively). A total of 19 genes are annotated only with an IEA code in yeast, and 29 in human. Concerning *List4*, using only IEA MF annotations does not lead to large changes in the value distribution when compared to *List1 *and *List2*. This suggests that these annotations are sufficient to capture intra-set pairwise similarity for such datasets. However, a significant number of missing values is observed in the yeast Pfam clan dataset, with 20 genes lacking any IEA MF annotations.

In summary, using customized weight lists for evidence codes in the IntelliGO measure is a useful way to highlight the contribution that certain types of annotations make to similarity values, as shown with the Pfam clan datasets and IEA MF annotations. However, this contribution clearly depends on the dataset and on the considered GO aspect. Other weight lists may be worth considering if there is special interest for certain ECs in certain datasets. In this study, we decided to continue our experiments with *List2 *since this weight list expresses the commonly shared view about the relative importance of ECs for gene annotation.

#### 2.4.4 Discriminating power

**Figure 5:**
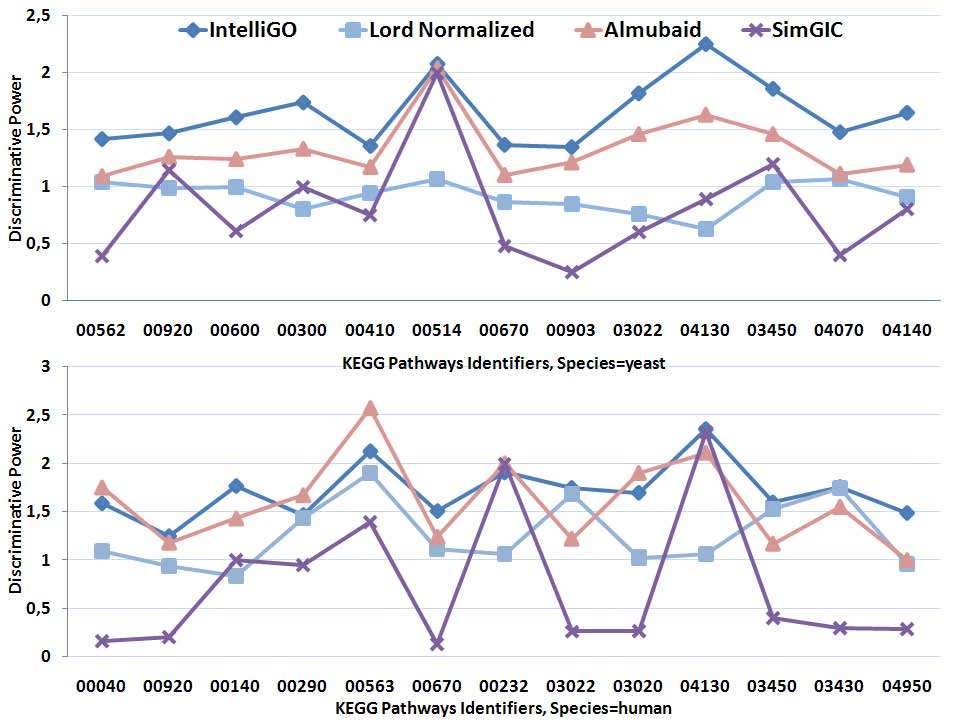
**Comparison of the inter-set discriminating power of four similarity measures using KEGG pathways and BP annotations**. The DP values obtained with the *IntelliGO*, Lord-normalized, Al-Mubaid, and SimGIC similarity measures are plotted for each KEGG pathway (top panel for yeast and bottom panel for human).

**Figure 6:**
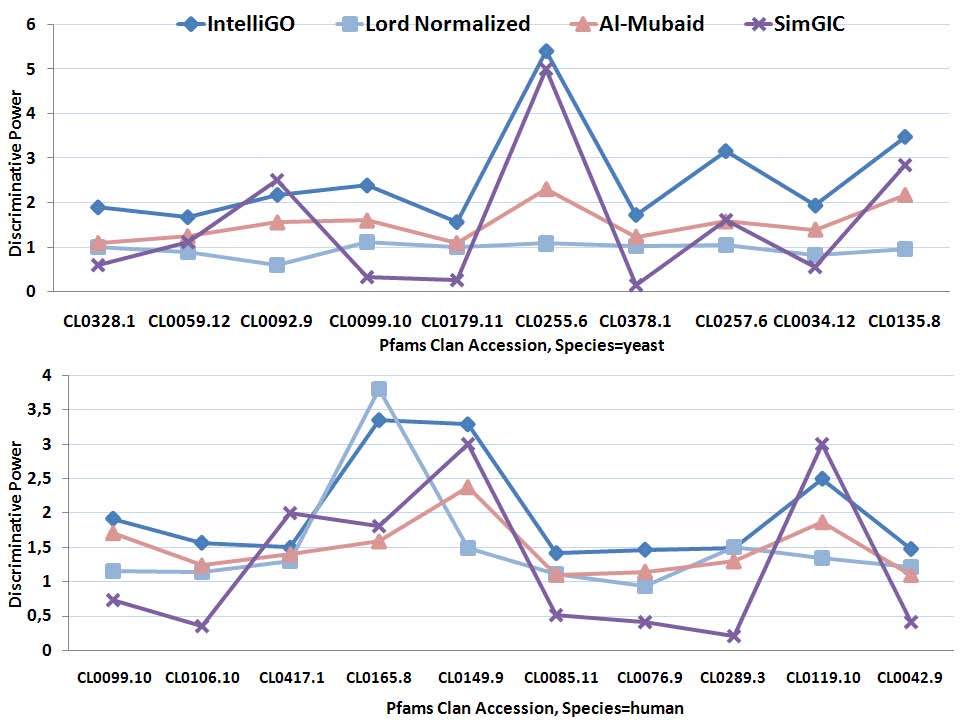
**Comparison of the inter-set discriminating power of four similarity measures using Pfam clans and MF annotations**. The DP values obtained with the *IntelliGO*, Lord-normalized, Al-Mubaid, and SimGIC similarity measures are plotted for each Pfam clan (yeast genes on top and human genes at bottom).

The third step of our evaluation consisted of testing the Discriminating Power (*DP *) of the *IntelliGO *similarity measure and comparing it with three other measures (Lord-normalized, Al-Mubaid, and SimGIC). The calculation of a discriminating power is introduced here to evaluate the ability of a similarity measure to distinguish between two functionally different sets of genes. The DP values for these three measures for the two benchmarking datasets are plotted in Figure 5 and 6. For the KEGG pathways and BP annotations (Figure 5), the *IntelliGO *similarity measure produces *DP *values greater than or equal to 1.3 for each tested pathway, with a maximum of 2.43 for the *hsa*04130 pathway. In contrast, the *DP *values obtained with the normalized Lord measure oscillate around 1.0 (especially for the yeast pathways), which is not desirable. The Al-Mubaid and SimGIC measures generate rather heterogeneous DP values ranging between 1.0 and 2.5, and 0.2 and 2.3, respectively. Such heterogeneity indicates that the discriminative power of these measures is not as robust as the *IntelliGO *measure.

The results are very similar and even more favorable for the *IntelliGO *similarity measure when using Pfam clans and MF annotations as the benchmarking dataset (Figure 6). In this case, all of the *IntelliGO DP *values are greater than 1.5, and give a maximum of 5.4 for Pfam clan *CL*0255.6. The three other measures give either very non discriminative values (e.g. Lord-normalized for yeast Pfam clans) or quite heterogeneous profiles (all other values). Overall, these results indicate that the *IntelliGO *similarity measure has a remarkable ability to discriminate between distinct sets of genes. This provides strong evidence that this measure will be useful in gene clustering experiments.

#### 2.4.5 Evaluation with the CESSM tool

A complementary evaluation was performed using the recent Collaborative Evaluation of GO-based Semantic Similarity Measures (CESSM) tool. This on-line tool [51] enables the comparison of a given measure with previously published measures on the basis of their correlation with sequence, Pfam, and Enzyme Classification similarities [52]. It uses a dataset of 13,430 protein pairs involving 1,039 proteins from various species. These protein pairs are characterized by their sequence similarity value, their number of common Pfam domains and their degree of relatedness in the Enzyme Classification, leading to the so-called *SeqSim*, *Pfam *and *ECC *metrics. Semantic similarity values, calculated with various existing methods, are then analyzed against these three biological similarity indicators. The user is invited to upload the values calculated for the dataset with his own semantic similarity measure. The CESSM tool processes these values and returns the corresponding graphs, a table displaying the Pearson correlation coefficients calculated using the user's measure as well as 11 other reference measures along with calculated resolution values for each measure.

We present in Table 4 only the results obtained for correlation coefficients as they are the most useful for comparison purposes. The values obtained with the *IntelliGO *measure using the MF annotation and including or excluding GO terms with IEA evidence codes are shown in the last column. When the whole GO annotation is considered (first three lines), the correlation coefficients range from 0.40 for the SeqSim metrics to 0.65 for ECC metrics. The value obtained with the ECC metrics is higher than all other values reported for this comparison, the best being the SimUI measure (0.63). For the Pfam and SeqSim metrics, the correlation coefficients obtained with the *IntelliGO *measure are lower than the best values obtained from five and seven other measures, respectively, the best values being obtained from the SimGIC measure (0.63 and 0.71, respectively). When IEA annotations are excluded (the final three lines), the IntelliGO correlation coefficients are lower for the ECC and Pfam metrics, as observed with most other measures, but slightly higher for the SeqSim metrics. This limited increase or absence of decrease is observed with two other measures (SimUI, JA), whereas much larger increases are seen for the three Max variants of the Resnick, Lord, and Jaccard methods (RM, LM, JM). Hence, it appears that in this evaluation, the *IntelliGO *measure gives correlation values that are intermediate between those obtained with poor (RA, RM, LA, LM, JA, JM) and high (SimGIC, SimUI, RB, LB, JB) performance methods.

**Table 4:** Evaluation results obtained with the CESSM evaluation tool.

Metrics		Method											
		
		SimGIC	SimUI	RA	RM	RB	LA	LM	LB	JA	JM	JB	IntelliGO
	ECC	0.62	0.63	0.39	0.45	0.60	0.42	0.45	0.64	0.34	0.36	0.56	0.65
	
	Pfam	0.63	0.61	0.44	0.18	0.57	0.44	0.18	0.56	0.33	0.12	0.49	0.48
	
All EC	SeqSim	0.71	0.59	0.50	0.12	0.66	0.46	0.12	0.60	0.29	0.10	0.54	0.40

	ECC	0.58	0.57	0.37	0.47	0.48	0.38	0.51	0.51	0.37	0.46	0.51	0.48
	
	Pfam	0.58	0.55	0.43	0.44	0.52	0.42	0.42	0.51	0.33	0.34	0.45	0.43
	
Non-IEA EC	SeqSim	0.66	0.59	0.46	0.48	0.65	0.41	0.40	0.59	0.31	0.36	0.52	0.43

Table 4: Pearson linear correlation coefficients are displayed for the ECC (Enzyme Classification Comparison), Pfam, and sequence similarity metrics (SeqSim). The *Molecular Function *GO annotation is used including (first three rows) or excluding (last three rows) annotation terms with IEA evidence codes. The column headings are listed as: SimUI: Union Intersection similarity; RA: Resnick Average; RM: Resnick Max; RB: Resnick Best match; LA: Lord Average; LM: Lord Max; LB: Lord Best match; JA: Jaccard Average; JM: Jaccard Max; JB: Jaccard Best match.

## 3 Discussion

Considering the growing number of semantic similarity measures, an important aspect of this study is the proposal of a method for estimating and comparing their performance. So far, rather heterogeneous and non-reproducible strategies have been used to validate new semantic similarity measures [9]. For example, it is generally assumed that gene products displaying sequence similarity should display similar MF annotations. This hypothesis was used by Lord *et al*. to evaluate their semantic similarity measure by exploring the correlation between gene annotation and sequence similarity in a set of human proteins [2]. They found a correlation between annotation and sequence similarity when using the MF aspect of GO annotations, and this was later confirmed by Schlicker *et al*. [28] using a different similarity measure. These authors also tested their similarity measure for clustering protein families from the Pfam database on the basis of their MF annotations. They showed that Pfam families with the same function did form rather well-defined clusters. In this frame of mind, the CESSM tool used in this study (Section 2.4.5) is a valuable initiative towards standardizing the evaluation of semantic similarity measures.

Another group of evaluation techniques relies on the hypothesis that genes displaying similar expression profile should share similar functions or participate in similar biological processes. This was used by Chabalier *et al*. [41] to validate their similarity measure. These authors were able to reconstitute networks of genes presenting high pairwise similarity based on BP annotations and to characterize at least some of these networks with a particular transcriptional behavior and/or some matching with relevant KEGG pathways.

Using pathways as established sets of genes displaying functional similarity has also become an accepted way to validate new similarity measures. The analyses performed by Guo *et al*. [35] showed that all pairs of proteins within KEGG human regulatory pathways have significantly higher similarity than expected by chance in terms of BP annotations. Wang *et al*. [29] and Al-Mubaid *et al*. [26] have tested their similarity measure on yeast genes belonging to some pathways extracted from the *Saccharomyces *Genome Database. In the former study, only MF annotations were considered and the authors' similarity measure led to clustering genes with similar functions within a pathway much more efficiently than a measure based on Resnik's similarity between GO terms. In the latter study, the values obtained for pairwise gene similarity using BP annotations within each studied pathway were also more consistent than those obtained with a measure based on Resnik's similarity.

In our study, two benchmarking datasets of KEGG pathways and Pfam clans were used to test the performance of the *IntelliGO *similarity measure. Expressions for intra-set similarity and inter-set discriminating power were defined to carry out this evaluation. The testing hypotheses used here are that genes in the same pathway or Pfam clan should share similar BP or MF annotations, respectively. These datasets contain 465 and 218 genes, respectively, which is less then the CESSM evaluation dataset (1,039 proteins). However, the calculation of intra-set and inter-set similarities led to 67,933 pairwise comparisons which is larger than in the CESSM dataset (13,340 protein pairs).

## 4 Conclusions and perspectives

This paper presents *IntelliGO*, a new vector-based semantic similarity measure for the functional comparison of genes. The *IntelliGO annotation *vector space model differs from others in both the weighting scheme used and the relationships defined between base vectors. The definition of this novel vector space model allows heterogeneous properties expressing the semantics of GO annotations (namely annotation frequency of GO terms, origin of GO annotations through evidence codes, and term-term relationships in the GO graph) to be integrated in a common framework. Moreover, the *IntelliGO *measure avoids some inconveniences encountered with other similarity measures such as the problem of aggregating at best term-term similarities. It also solves rigorously the problem of multiple depth values for GO terms in the GO rDAG structure. Furthermore, the effect of annotation heterogeneity across species is reduced when comparing genes within a given species thanks to the use of IAF coefficients which are constrained to the given species. Our results show that the *IntelliGO *similarity measure is robust since it copes with the variability of gene annotations in each considered set of genes, thereby providing consistent results such as an intra-set similarity value of at least 0.50 and a discriminative power of at least 1.3. Moreover, it has been shown that the *IntelliGO *similarity measure can use ECs to estimate the relative contributions of GO annotations to gene functional similarity. In future work, we intend to use our similarity measure in clustering experiments using hierarchical and *K-means *clustering of our benchmarking datasets. We will also test co-clustering approaches to compare functional clustering using *IntelliGO *with differential expression profiles [24,53].

## 5 Methods

The C++ programming language was used for developing all programs. The extraction of the *LCAs *and the *SPLs *of pairs of GO terms was performed by querying the GO relational database with the *AmiGO *tool [54].

### 5.1 Reference Similarity Measures

The four measures compared in our evaluation were implemented using the following definitions. Let *g*_1 _and *g*_2 _be two gene products represented by collections of GO terms *g*_1_={*t*_1,1_, ..., *t*_1,*i*_, ..., *t*_1,*n*_} and *g*_2 _= {*t*_2,1_, ..., *t*_2,*i*_, ..., *t*_2,*m*_}. The first measure is Lord's similarity measure [2], which is based on Resnik's pairwise term similarity. For each pair of terms, *t_i _*and *t_j_*, the Resnik measure is defined as the information content (IC) of their *LCA*:

(21) $$SIM_{Resnik}(t_{i},t_{j})=IC(LCA(t_{i},t_{j})).$$

Then, the Lord similarity measure between *g*_1 _and *g*_2 _is calculated as the average of the Resnik similarity values obtained for all pairs of annotation terms:

(22) $$SIM_{Lord}(g_{1},g_{2})=\frac{\sum_{i=1}^{n}\sum_{j=1}^{m}SIM_{Resnik}(t_{1,i},t_{2,j})}{n*m}.$$

Because this measure yields values greater than 1.0, we normalize the values obtained for a set of genes or for a collection of sets by dividing by the maximal value.

The second measure used here was introduced by Al-Mubaid *et al*. [31]. This method first calculates the shortest path length (PL) matrix between all pairs of GO terms annotating the two genes, i.e. PL (*t*_1,*i*_,*t*_2,*j*_), ∀i ∈[1,n], ∀j ∈[1,m]. It then calculates the average of all PL values in the matrix, which represents the average PL between the two gene products:

(23) $$PL(g_{1},g_{2})=\frac{\sum_{i=1}^{n}\sum_{j=1}^{m}PL(t_{1,i},t_{2,j})}{n*m}.$$

Finally, a transfer function is applied to this PL value to convert it into a similarity value. As this similarity monotonically decreases when the PL increases, the similarity value is obtained by:

(24) $$SIM_{Al-Mubaid}(g_{1},g_{2})=e^{-f*PL(g_{1},g_{2})},$$

with *f *= 0.2 according to the authors.

The third measure used here is the classical weighted-cosine measure, whereby each gene is represented by its annotation vector in an orthogonal VSM. Each component represents a GO term and is weighted by its own IAF value (*w_i _*= *IAF *(*t_i_*)) if the term annotates the gene, otherwise the weight is set to 0.0. Then, the weighted-cosine measure is defined in (20) but with the classical dot product expression in which ${\vec{g}}_{1}\cdot {\vec{g}}_{2}=\sum_{i}w_{i}^{2}$, for all terms *t_i _*present in both annotation vectors.

The last measure used here is SimGIC (Graph Information Content), which is also known as the Weighted Jaccard measure [36]. This measure is available in the *csbl.go *package within R Bioconductor [55], [56]. Given two gene products *g*_1 _and *g*_2 _represented by their two extended annotation sets (terms plus ancestors), the semantic similarity between these two gene products is calculated as the ratio between the sum of the information contents of GO terms in the intersection and the sum of the information contents of GO terms in the union:

(25) $$SimGIC(g_{1},g_{2})=\frac{\sum_{t\in g_{1}\cap g_{2}}IC(t)}{\sum_{t\in g_{1}\int g_{2}}IC(t)}.$$

### 5.2 Intra-Set and Inter-Set Similarity

Consider *S*, a collection of sets of genes where *S *= {*S*_1_, *S*_2_, ..., *S_i_*} (a set *S_k _*can be a KEGG pathway or a Pfam clan). For each set *S_k_*, let {*g*_*k*1_, *g*_*k*2_, ....., *g_kn_*} be the set of *n *genes comprised in *S_k_*. Let *Sim*(*g*, *h*) be a similarity measure between genes g and h. The intra-set similarity value is defined for a given set of genes *S_k _*by:

(26) $$Intra_{-}Set_{-}Sim(S_{k})=\frac{\sum_{i=1}^{n}\sum_{j=1}^{n}Sim(g_{ki},g_{kj})}{n^{2}}.$$

For two sets of genes *S_k _*and *S_l _*composed of *n *and *m *genes respectively, we define the inter-set similarity value by:

(27) $$Inter_{-}Set_{-}Sim(S_{k},S_{l})=\frac{\sum_{i=1}^{n}\sum_{j=1}^{m}Sim(g_{ki},g_{lj})}{n*m}.$$

Note that when the *Sim *function takes values in the interval 0[1], so do the *Intra_Set_Sim *and *Inter_Set_Sim *functions. Finally, for a given collection *S *composed of *p *sets of genes, the discriminative power of the semantic similarity measure *Sim *with respect to a given set *S_k _*in *S *will be defined as:

(28) $$DP_{Sim}(S_{k})=\frac{\left(p-1)Intra_{-}Set_{-}Sim(S_{k}\right)}{\sum_{i=1,i\neq k}^{p}Inter_{-}Set_{-}Sim(S_{k},S_{i})}.$$

## Authors' contributions

SB, MD and MS designed the IntelliGO similarity measure. SB developed and implemented the method. MD and MS evaluated the biological results. MD, MS, OP and AN supervised the study, making significant contributions. SB, MD and MS wrote the paper. All authors proofread and approved the final manuscript.
